# Quality indicators in colonoscopy: observational study in a supplementary health system

**DOI:** 10.1590/acb371106

**Published:** 2023-01-06

**Authors:** Rogerio Kuga, Marcio Roberto Facanali, Everson Luiz de Almeida Artifon

**Affiliations:** 1 MD, MSc. Universidade de São Paulo – Postgraduate Program in Anesthesiology, Surgical Sciences and Perioperative Medicine – School of Medicine – São Paulo (SP), Brazil; Hospital Samaritano de São Paulo , Americas Medical Services, UnitedHealth Group Brasil – São Paulo (SP), Brazil.; 2 MD. Universidade de São Paulo – Gastroenterology Department – Hospital das Clínicas – School of Medicine – São Paulo (SP), Brazil.; 3 MD, MSc, PhD. Universidade de São Paulo – Surgery Department – Hospital das Clínicas – School of Medicine – São Paulo (SP), Brazil

**Keywords:** Quality Indicators, Health Care, Colonoscopy, Adenomatous Polyps, Colonic Neoplasms

## Abstract

**Purpose:**

Colorectal cancer is responsible for 9.4% of cancer deaths, and low polyp detection rate and cecal intubation rate increase the risks of interval colorectal cancer. Despite several population studies that address colonoscopy quality measures, there is still a shortage of these studies in Latin America. The aim of this study was to assess quality indicators in colonoscopy, enabling future strategies to improve colorectal cancer prevention.

**Methods:**

An observational retrospective study, in which all colonoscopies performed in 11 hospitals were evaluated through a review of medical records. Information such as procedure indication, colorectal polyp detection rate, cecal intubation rate, quality of colonic preparation, and immediate adverse events were collected and analyzed.

**Results:**

In 17,448 colonoscopies performed by 86 endoscopists, 57.9% were in patients aged 50 to 74 years old. Colon preparation was adequate in 94.4% procedures, with rates of cecal intubation and polyp detection of 94 and 36.6%, respectively. Acute adverse events occurred in 0.2%. In 53.9%, high-definition imaging equipment was used. The procedure location, colon preparation and high-definition equipment influenced polyp detection rates (p < 0.001).

**Conclusions:**

The extraction and analysis of electronic medical records showed that there are opportunities for improvement in colonoscopy quality indicators in the participating hospitals.

## Introduction

According to the World Health Organization, in 2019, cancer is among the first or second cause of death in more than 110 countries and, of these, colorectal cancer is the third most diagnosed and the second cause of death among all cancers, responsible for 9.4% of cancer deaths worldwide^1^. In Brazil, except for non-melanoma skin cancer, colorectal cancer is the second most common cancer in both genders, according to data from 2020 from the National Cancer Institute (INCA).

Colorectal cancer screening is based on two distinct principles: early detection, which enables treatment before it reaches an incurable state, and preventive colorectal cancer screening, which detects and removes precursor lesions, such as adenomas^2^.

So far, there are no structured colorectal cancer screening programs in Brazil and, despite the global consensus on their importance, as in most parts of the world, they are offered to the minority of the population through colonoscopy and in a timely manner^3^.

In several studies^4^^-^ 7, low polyp detection rate (PDR), as well as low cecal intubation rate (CIR), has been observed to increase the risk of interval colorectal cancer. Population studies that addressed the quality of colonoscopy, as in Germany^8^, Austria^9^ and the Netherlands^10^, showed rates above 90% of CIR and more than 20% of adenoma detection rate (ADR), whereas in Italy^11^ the CIR was 80% and in France^12^ the ADR was below 20%.

There is a shortage of quality measures studies on colonoscopy in Latin America, especially in Brazil. Thus, the objective of this study was to evaluate the main quality indicators in colonoscopy, allowing future strategies to improve colorectal cancer prevention and health promotion.

## Methods

### Ethical approval

This project was approved by the Ethics and Research Committee of Hospital Samaritano de São Paulo (CAAE 57390722.9.0000.5487).

### Study design and population

A multicentric retrospective cohort was performed, based on data extraction from the Picture Archiving and Communication System — Radiology Information System (PACS-RIS) electronic endoscopic reporting system (CareStream Health, Rochester, NY, United States of America). All colonoscopies performed between January 2021 and December 2021, in 11 hospital units of a private supplementary health system in Brazil, distributed in seven cities in two Brazilian states, were evaluated. These hospital units were identified by the letters of the Roman alphabet, from A to K.

### Inclusion and exclusion criteria

The inclusion criteria were:

- Colonoscopies in patients 18 years of age or older;
- Colonoscopies in outpatients or inpatients;
- Elective or urgent colonoscopy;
- Colonoscopies that presented complete information in the reporting system.

All colonoscopies in patients younger than 18 years of age were excluded, as well as those with incomplete information in the reporting system.

### Instruments and procedures

#### Colon preparation

The Boston scale was used to assess colon preparation^13^, in which each of the three colon segments (right colon, transverse colon, and left colon and rectum) is scored from 0 to 3 (0 = inadequate, 1 = poor, 2 = good, and 3 = excellent), considering the sum of scores greater than or equal to 6 as satisfactory colon preparation.

#### Cecal intubation and cecal intubation rate

Cecal intubation was considered when the tip of the colonoscope passed the ileocecal valve, making it possible to identify the appendicular ostium and complete evaluation of the cecum. CIR was defined as the percentage of all colonoscopies in which the cecum was reached.

#### Polyp detection and polyp detection rate

Polyp detection was considered when a polyp was detected in the colon or rectum during colonoscopy. PDR was considered as the percentage of all colonoscopies in which at least one colorectal polyp was detected.

### Acute adverse events

The following situations were defined as acute or immediate adverse events:

- Acute hemorrhage: when there is a need for endoscopic intervention, such as the use of vasoactive substances, clips or coagulation to contain the hemorrhage, or even the need for surgical intervention;
- erforation: when there is transmural injury of the organ with communication of the organ’s lumen with the peritoneal cavity, regardless of whether treated endoscopically or surgically;
- Broncho aspiration: defined when there is gastric content in the lower airways through regurgitation of gastric content during colonoscopy;
- Anaphylaxis: when there is a hypersensitivity reaction during sedation or colonoscopy that requires medical intervention, such as administration of epinephrine and antiallergic or corticosteroids;
- Cardiorespiratory event: any cardiac or respiratory change that requires the interruption of colonoscopy and medical maneuvers, such as in severe arrhythmias, respiratory depression requiring orotracheal intubation triggered as a result of the procedure.

### Other analyzed variables

Other variables described ahead were also evaluated:

- Indications for performing colonoscopy;
- Time of training and qualification in endoscopy in years of endoscopists;
- Use of high-definition image endoscopes, defined when there are more than 400k pixels of image definition in the image capture by the colonoscope.

All colonoscopies were performed under sedation with midazolam, fentanyl, and propofol by the anesthesiology team with multiparametric monitoring (heart rate, cardiac monitoring, non-invasive blood pressure, and pulse oximetry) of the patient.

### Statistical analysis

All analyses were performed using the R statistical software (R Core Team, 2018).

Depending on the need and the use of study variables, descriptive analysis of sample size (N), standard deviation (SD) was used; minimum, 1st quartile, median, 3rd quartile, maximum and interquartile range (IQ). For the analysis between the variables of the sample, the Inferential analysis was used, applying Pearson’s χ^2^ test with interpretation of the p-value of the test as statistically significant when p is less than 0.05. In some situations, Fisher’s exact test was performed, which can be interpreted in the same way. For non-parametric tests, the Shapiro-Wilk’s normality test and the Kruskal-Wallis’ test were used, both also interpreted when there is a level of statistical significance if p < 0.05.

Regarding the tests to analyze the relationships of interest between two variables, the χ^2^ test or Fisher’s exact test were performed in order to study the association between two categorical variables; and the Wilcoxon’s test or the Kruskal-Wallis’ test to study the relationship between a numerical and a categorical variable when the categorical has two or at least three categories, respectively.

The statistical study also used logistic regression models in order to identify factors associated with the presence of polyp, comparing the variables by the *odds ratio* (OR), with the statistical significance of 5%, if the p-value of any of the variable’s categories was lower than 0.05. The confidence interval was 95% for the OR.

## Results

Between January and December 2021, 17,448 colonoscopy exams were performed in 11 private hospital units, by 86 endoscopists. Among the patients, 61.3% were female and 38.7% were male. Most patients underwent the examination electively (85.3%), 14.4% of the patients underwent the examination inpatients, and only 41 patients (0.2%) underwent the examination on an emergency basis. In 39.3% of cases, patients had never had a colonoscopy before.

Screening for colorectal cancer was the main indication for colonoscopy, accounting for more than half of all indications (Table 1).

**Table 1:** Main indications for colonoscopy.

Colonoscopy Indications	Frequency (n)	Percentage (%)
Anemia	184	1.1
Constipation	318	1.8
Diarrhea	984	5.6
Abdominal pain	1.636	9.4
Family history of colorectal cancer	159	0.9
Colorectal cancer screening	9.650	55.3
Colorectal bleeding	1.153	6.6
Colorectal cancer surveillance	2.334	13.4
Others	1.030	5.9
Total	17.448	100

**Figure 1:**
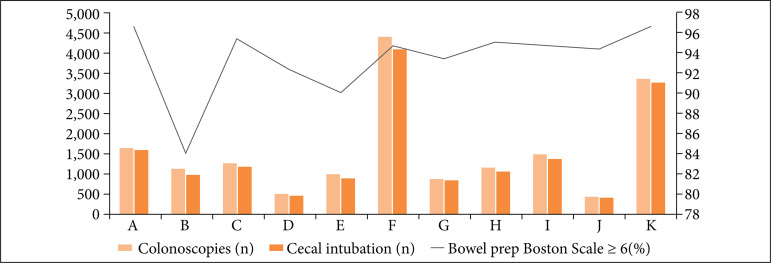
Number of colonoscopies performed, cecal intubation, and bowel preparation rate per hospital unit.

Bowel preparation was satisfactory in 94.4% of all patients, with rates above 90% in all hospital units, except for Hospital B, where satisfactory preparation was achieved in 84.3% of colonoscopies (Fig. 1). The cecum was intubated in 94% of all colonoscopies. Of the 1,045 colonoscopies that did not reach the cecum, 49.3% were due to inadequate bowel preparation, and 50.7% due to malignant or benign stenosis, megacolon, technical difficulty, intra-abdominal post-surgical adhesions, and miscellaneous other causes. The CIR is an indicator of colonoscopy quality directly influenced by the quality of colon preparation, with a lower CIR being observed in Hospital B and better in Hospital K, with statistical significance (p < 0.001, χ^2^).

**Figure 2:**
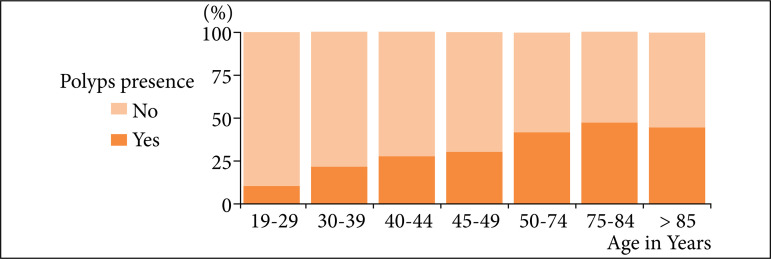
Frequency of polyp detection rate by age group.

**Figure 3:**
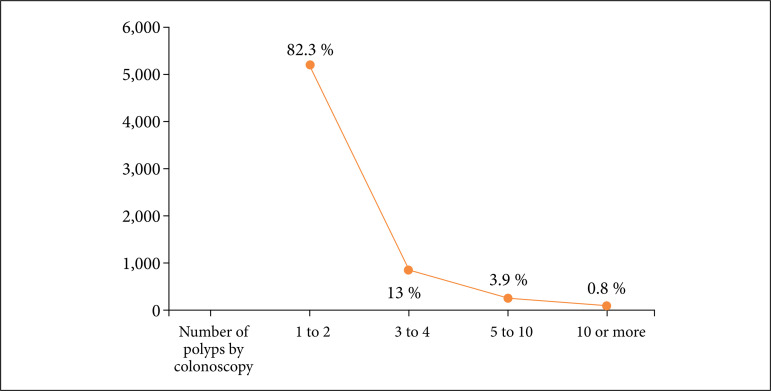
Distribution of frequency and percentage of the number of polyps in each colonoscopy.

Colon polyps were detected in 6,389 colonoscopies, yielding an overall PDR of 36.6%. Males, despite being less frequent in the sample, had a higher PDR (42.8%) compared to females (32.6%), with statistical significance by Fisher’s exact test (p < 0.001). It was also observed that there was a correlation between the PDR and the age group, with statistical significance in the increase in the PDR with increasing age group. Young patients, 18 to 29 years old, had a PDR of 10.3%, as shown in Fig. 2, and 50 years and older had PDR over 40%. The number of polyps detected during colonoscopy were stratified, and most of the procedures (82.3%) found one or two polyps (Fig. 3).

**Figure 4:**
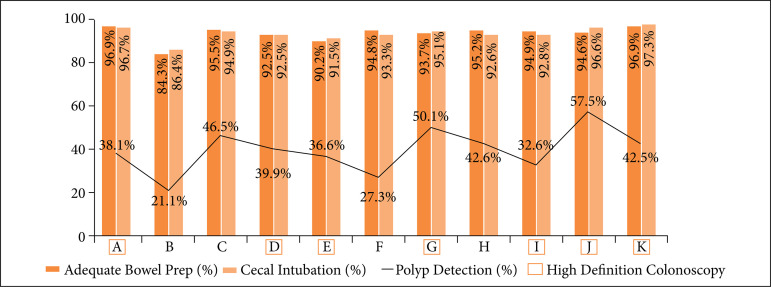
Correlation of polyp detection rate with bowel preparation, cecal intubation,and presence of high-definition colonoscopes per hospital.

The performance of colonoscopies using high-definition endoscopic equipment influenced the finding of colon polyps, significantly increasing the PDR (p < 0.001, Fisher). The correlation of the indicators PDR and CIR showed statistical significance, demonstrating the greater detection of polyps the greater rate of cecal intubation (37.9% detection rate of polyps with cecal intubation *vs*. 84% no polyp detection when the cecum is not reached, p < 0.001, Fisher). There was also this relationship on the quality of colon preparation according to the Boston scale, *i.e.*, in which colon cleansing is more appropriate, colon and rectal polyps are diagnosed more often and, consequently, the detection rate of polyps increases (p < 0.001, Fisher). The comparative analysis of the PDR and hospital variables showed that there is a statistically significant difference in colon polyp detection in relation to the place where the procedure was performed (p < 0.001, χ^2^)–Hospital B had the lowest PDR (21.1%) and Hospital J the highest one (57.5%) (Fig. 4).

The number of colonoscopy procedures performed with high-definition endoscopic imaging equipment, according to the availability of this technology in the participating hospitals, corresponded to the majority of procedures performed (53.9%).

In the total sample of 17,448 colonoscopies, the performance of the first colonoscopy, that is, the absence of previous colonoscopies since birth, was not a variable that influenced the PDR (p = 0.748), with this group obtaining a detection rate of 36.5%, similar to the overall rate.

In 32 colonoscopies, there were acute adverse events, representing an overall rate of immediate adverse events of 0.2%, with hemorrhage after endoscopic polyp resection the most common cause in 14 of the 32 colonoscopies, representing 0.08% of all colonoscopies performed (Table 2).

**Table 2:** Acute adverse events during colonoscopy.

Acute adverse event	Frequency (n)	Percentage of all Colonoscopies (%)
Bleeding	14	0.08
Perforation	5	0.03
Bronchoaspiration	3	0.02
Anaphylaxis	1	0.01
Cardiorespiratory	3	0.02
Others	6	0.03
Total	32	0.2

**Figure 5:**
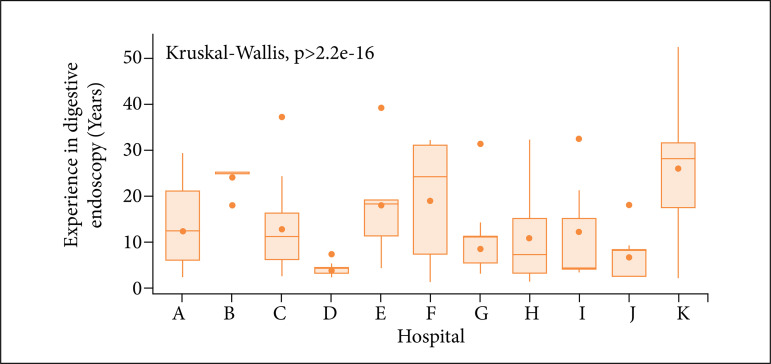
Boxplot of the variable years of training in digestive endoscopy by the hospital unit.

The mean experience in gastrointestinal endoscopy of the 86 physicians who performed colonoscopies after completing the specialization course was 13 years (ranging from 1 to 52 years), with a median of 9.5 years. There was a statistically significant difference in the distribution of the experience variable of physicians, which tend to be shorter in hospitals D, G and J and longer in hospitals B and K, with greater variation in time in Hospital F, as shown in Fig. 5.

The PDR among endoscopists presented a mean and median of 42.8 and 42.7%, respectively, and, interestingly, physicians with less years of experience in digestive endoscopy had higher PDR (p < 0.001). However, endoscopists with longer specialized training did not influence the CIR (p = 0.484).

## Discussion

This is the first Brazilian multicenter study in a private supplementary health system that evaluates quality indicators in colonoscopy. In this study, we retrospectively analyzed data compiled from 11 private hospital units, demonstrating that, although there is some variability between variables and indicators, in general there are good quality and safety in performing colonoscopies in these institutions.

Colonoscopy has a great role in the prevention of colorectal cancer, and the colonoscopy quality measures evaluated in this exam–colon preparation according to Boston scale ≥ 6, CIR, PDR–are important due to the interrelationship between each other in this process, whose objective is to carry out a procedure in good conditions, with quality and safety, and avoiding unnecessary expenses.

However, an important initial step in this process is the indication of colonoscopy. Performing the procedure with adequate and precise indication increases the sensitivity of the procedure, avoids wasting resources in the health system and reduces the patient’s exposure to the risks inherent to the procedure^14^. One of the indications for colonoscopy accepted as appropriate would be in the situation screening and surveillance of colorectal cancer in asymptomatic patients or not, as well as follow-up after colon polypectomy in previous colonoscopy, and these situations are nowadays the most frequently encountered in endoscopy services.

Another frequent reason for indication would be in situations of suspicion or confirmation of gastrointestinal bleeding, depending on the clinical presentation of the patient, such as anemia, presence of occult blood in the feces, or explicit visual evidence of gastrointestinal bleeding, such as melena, and hematochezia. Other indications such as investigation of diarrhea, abdominal pain, suspicion, or follow-up of inflammatory bowel disease, altered image on radiological examination such as colonic parietal thickening, colon involvement due to adjacent pathologies, such as endometriosis, and colonoscopy with therapeutic intent defined as dilation, hemostasis, resection, decompression, tattooing, among others, are also reasons for performing the exam and considered appropriate indications for the procedure.

In the present study, 94.1% of the indications for colonoscopy were considered adequate, being higher than the minimum standard of ≥ 85% recommended by the European Society of Gastrointestinal Endoscopy (ESGE) and slightly below the ideal (≥ 95%)^15^.

The quality of colon preparation, which in the present study was classified using the Boston scale^13^^,^ 16, is the most used colonoscopy quality index in the literature, which classifies colon preparation as adequate when the score is ≥ 6.

Information on colon preparation conditions in the colonoscopy report is desirable, either by medical description of poor, inadequate, satisfactory or adequate preparation or by numerical ratings by score. Among the total of 17.448 colonoscopies, 16.474 (94.4%) were considered ≥ 6 by the Boston scale. By ESGE, the minimum standard is ≥ 90%, and the desired goal is ≥ 95% of colon preparations with a score ≥ 6.

The quality of colon preparation varied between hospitals–Hospital B achieved the lowest indicator. This hospital is the only one among the 11 locations that has two options for colon preparation, depending on the patient’s choice or the requesting physician’s indication, which can be performed with 10% mannitol solution or sodium picosulfate. In the sample, 1,149 colonoscopies were performed at Hospital B, and information on the type of solution used was not obtained, which is an important bias that prevented sample homogeneity in this variable. In the study carried out by Miki Jr. *et al*.^17^, 90% of the patients who were prepared with mannitol had 90% adequate colon preparation, while only 50% of users who used sodium picosulfate had adequate colon preparation. The Boston scale ≥ 6 points in 84.3% at Hospital B impacted the polyp detection rate (21.1%) and the cecal intubation rate (86.4%). This finding reinforces what was observed by Froehlich et al.^18^ about the direct impact of the quality of colon preparation on these indicators.

The use of the CIR as a colonoscopy quality measure is due to the ability of colonoscopy to evaluate the entire colorectal mucosa, and only when reaching the cecum can this evaluation be achieved. The overall CIR in this study was 94%. The minimum standard by the ESGE is to be ≥ 90%, with the goal of achieving an index ≥ 95%^15^. The American Society of Gastrointestinal Endoscopy (ASGE) recommends a CIR ≥ 90% overall for all colonoscopies performed as a goal, and index ≥ 95% in the situation of indication for colorectal cancer screening^19^.

The success in achieving high rates of CIR is not only related to the quality of the colon preparation, as mentioned before, but also the ability to technique and skill of the endoscopist, as the procedure is operator-dependent and the examination is considered to be complete when the cecum is reached and, thus, all segments of the colon will be subject to endoscopic evaluation.

As the reason for the failure to reach the cecum, almost half were attributed to inadequate colon preparation (49.3%). Failure to reach the cecum generates a feeling of frustration in the entire team involved in patient care and has repercussions on their experience, as well as resulting in new future costs for the eventual rescheduling of a new colonoscopy procedure or organization of another method for the investigation of the colon. Not only the emotional and frustrating impacts of not performing the complete procedure, as well as the feeling of wasted time and resources, there is a loss of opportunity for diagnosis of both advanced and initial tumor lesions that would be amenable to endoscopic treatment by colonoscopy.

In the study performed by Baxter *et al*.^4^, endoscopists with a cecal intubation rate ≥ 95% have a lower 36-month interval colon cancer rate compared with physicians with a cecal intubation rate of less than 80%. On the other hand, in the present study, despite using the hypothesis that endoscopists with longer training in the area and with more experience would be more successful in reaching the cecum and would reach a higher rate of cecal intubation, the analysis of these variables did not reveal a statistically significant association. Additionally, the logistic regression model did not demonstrate statistical significance between the rate of cecal intubation and the years of training in digestive endoscopy [p = 0.498; OR = 0.998 (95% interval of confidence–95%CI 0.992–1.004)].

The overall PDR in the 17,448 colonoscopies was 36.6%. The ESGE wants this rate to be ≥ 40%^15^. The PDR ranged from 21.1 (Hospital B) to 57.5% (Hospital J), and despite the overall rate of the sample present at a reasonable level (36.6%), there is heterogeneity in the assessment, both in the view of the medical group per hospital and individually.

The medical variability in the PDR is of great concern, as it impacts not only the adenoma detection rate, but also directly the risk of interval colorectal cancer^20^^,^ 21. Action plans to improve this quality index can be performed, not only in correcting the steps of the colonoscopy process, but also through education and regular feedback with the hospital or physician group with the aim of increasing the detection rates of polyps and adenomas, identifying and training physicians with low performance^22^.

Additionally, it is believed that not only the feedback, but also the knowledge of the endoscopist is being observed, measured, and monitored, and it can change its attitude during the colonoscopic examination and reflect on the improvement and increase of this index, a phenomenon called the Hawthorn effect^23^. As a hypothesis, as well as for the rate of cecal intubation, it was assumed that the time of experience and years after training in gastrointestinal endoscopy would have a positive impact on the detection rate of polyps. It was observed that this correlation existed in the present study, but in an inverse way, at a statistical significance of 5%; when increasing by one year the years of formation of the endoscopist who performs the procedure, the chance of polyp detection is reduced by almost 2% [p < 0.001; OR = 0.984 (95%CI 0.982–0.987)].

The existence of high-definition imaging endoscopic equipment was variable captured and analyzed, in which in its presence the PDR was 40.8% and, in its absence, 31.7% (p < 0.001, Fisher). The logistic regression model also revealed a direct correlation in the presence of high-definition imaging equipment and a higher PDR [p < 0.001; OR = 1.483 (95%CI 1.393–1.578)]. This outcome was observed in the meta-analysis by Subramanian *et al*.^24^, which revealed a 3.8% increase in polyp detection when high-definition imaging equipment was used (95%CI 1–6.7). With the intention of increasing the PDR^25^, some recommendations such as performing maneuvers with the colonoscope such as retroflexion in the right colon, changing the patient’s position during the examination, using devices at the end of the colonoscope to improve the colorectal mucosa exposure or chromoendoscopy were not captured in the database, and it is not possible to evaluate these variables in this sample, neither to say whether they were performed or not.

One of the limitations of the present study was the failure to capture the anatomopathological results of the colorectal polyps removed to calculate the ADR. Regular and systematic anatomopathological checks, as well as manual insertion into a spreadsheet for later calculation, are an arduous and time-consuming task, whether in the administrative sector or the endoscopist doctor, which means that in day-to-day practice this index is not evaluated.

Due to this practical difficulty, the PDR was used instead, due to the correlation with the ADR observed by Francis *et al*.^26^, in which 40% of the PDR is correlated to 25% of ADR in this study. However, the use of the PDR as a key performance indicator (KPI) in colonoscopy is subject to criticism, often related to the medical remuneration model, when this payment is performed per procedure, as in fee-for-service model. The individual or group is subject to an excessive increase in this KPI, and the increase in the PDR may not correlate with the increase in the ADR, which, in turn, is also subject to criticism, as the finding and diagnosis of only one adenomatous polyp counts for this indicator. In this situation, when the endoscopist is aware of this measurement as an individual or group, it may occur that, after detecting an adenoma-like polyp during colonoscopy, the endoscopist practically no longer pays attention to looking for other polyps and removes quickly the colonoscope without examining the rest of the colon properly. This behavior is called *one and done*, that is, when it finds a polyp with an adenomatous aspect, it ends the work and does not examine the rest of the colon in detail^19^.

In place of the ADR, to mitigate this situation, there are authors who recommend the use of the adenoma per colonoscopy (APC). In this colonoscopy quality measure, the number of diagnosed and resected adenomas counts for the index, and not only the presence of adenoma, even if only one^27^. In the present study, for those patients who were identified at least one polyp during colonoscopy, the number of polyps observed was stratified, with the predominance of one or two polyps per colonoscopy (n = 5,256; 82.3%). Such a finding of the number of polyps by colonoscopy could infer the rate of APC, but there is no such correlation described in the literature. Some variables can influence the PDR and the development of colorectal cancer, such as age and male gender^28^. In this series, it was demonstrated that ages over 50 years and males had a PDR higher than the target recommended by the ESGE of > 40%, with an estimated ADR in accordance with the estimated and achievable of 25%^29^^,^ 30.

In addition to the limitation of the present study of not evaluating the ADR due to the difficulty in accessing and tabulating the anatomopathological results, the time taken to remove the colonoscope was not measured. A detailed evaluation of the colonic mucosa during the colonoscopic examination is performed during the removal of the device, and the time taken to remove the device is considered a quality index in colonoscopy, since the withdrawal time to carefully inspect the colonic mucosa is related to greater diagnosis of colorectal polyp^31^. This inspection time is computed from the reach of the cecum, and the removal time to the rectum must be at least 6 minutes, which is related to higher PDR and ADR^10^, not considering the time of biopsy, polypectomy or other therapeutic procedure. However, not only is the goal of a device withdrawal time of ≥ 6 minutes desired, but also the technique employed by the colonoscopist, such as careful analysis behind the colonic folds, cleaning and aspiration of small fecal residues, and adequate distention of the colon. The measurement and daily basis monitoring, exam by exam, of the colonoscopy withdrawal time is laborious, and there is no automatic tool to capture this data, requiring the support and participation of the room assistant. The capture of the device removal time can be used as an improvement action plan for endoscopists who have low performance in ADR (< 25%) or PDR (< 40%)^32^.

Regarding the most common and important adverse events described in the literature, colon perforation, post-procedure hemorrhage and death related to the exam are mentioned, being estimated at 0.05, 0.26 and 0.029%, respectively^33^. In another series, Kothari *et al*.^34^ showed that the overall incidence of perforation in more than 10 million colonoscopies was 0.06%, with a lower incidence in diagnostic-only colonoscopies and higher when endoscopic therapy is associated with the procedure. The total incidence of hemorrhage was 0.24%, being more related to procedures in which endoscopic resection was performed, such as polypectomy. The mortality rate specifically associated with colonoscopy is fortunately a rare event (0.007%), and the total incidence of mortality is 0.03%. The maximum frequency expected in the literature for complications or late adverse events at seven and 30 days, whether or not associated with readmission, is ≤ 0.5%^15^^,^ 35.

In this sample, there were 32 immediate or acute complications (0.2%). Fourteen post-endoscopic resection hemorrhages (0.08%), five colon perforations (0.03%) and no mortality during the procedure. Multivariate analysis with logistic regression did not reveal statistical significance, with no increase or decrease in acute or immediate complications with time since endoscopist training (p = 0.072; OR = 0.977; 95%CI 0.952–1.001).

As these are data captured from the database from completing the questionnaire at the end of the exam, there is no information on complications at seven and 30 days, such as hemorrhage or late perforation after endoscopic resection and readmission due to an adverse event related to colonoscopy. Obtaining data on late colonoscopy complications from reporting systems was a limitation of the present study, as it was for Bretthauer *et al*.^36^.

## Conclusions

There was a direct correlation between the quality of colon preparation (Boston scale ≥ 6), higher CIR and higher PDR of the colon and rectum.

Not only the place where the procedure was performed (hospital unit or medical group), but also the individual physician influenced the main colonoscopy quality measures (CIR and PDR), with variability in these rates.

The time after specialized medical training in gastrointestinal endoscopy influenced the PDR, which was higher in the younger physicians, but the CIR and the rate of adverse events were not influenced by this variable.

Colonoscopy performed with high-definition imaging equipment influenced the increase in PDR.

The data revealed that there is room for improvement in the performance of this procedure in order to increase the quality indicators and colorectal prevention in these participating hospitals.
